# Isolation of viable but nonculturable *Vibrio cholerae* O1 from environmental water samples in Kolkata, India, in a culturable state

**DOI:** 10.1002/mbo3.164

**Published:** 2014-02-18

**Authors:** Mitsutoshi Senoh, Jayeeta Ghosh-Banerjee, Tamaki Mizuno, Sumio Shinoda, Shin-ichi Miyoshi, Takashi Hamabata, G Balakrish Nair, Yoshifumi Takeda

**Affiliations:** 1 Collaborative Research Center of Okayama University for Infectious Diseases in India, Okayama UniversityKolkata, India; 2 National Institute of Cholera and Enteric DiseasesKolkata, India; 3 Graduate School of Medicine, Dentistry and Pharmaceutical Sciences, Okayama UniversityOkayama, Japan; 4 Research Institute, National Center for Global Health and MedicineShinjuku, Tokyo, Japan; 5 Translational Health Science and Technology InstituteHaryana, India

**Keywords:** Factor converting VBNC into culturable, VBNC*Vibrio cholerae*

## Abstract

Previously, we reported that viable but nonculturable (VBNC) *Vibrio cholerae* was converted into a culturable state by coculture with several eukaryotic cell lines including HT-29 cells. In this study, we found that a factor converting VBNC *V. cholerae* into a culturable state (FCVC) existed in cell extracts of eukaryotic cells. FCVC was nondialyzable, proteinase K-sensitive, and stable to heating at <60°C for 5 min. We prepared thiosulfate citrate bile salts sucrose (TCBS) plates with FCVC (F-TCBS plates). After confirming that VBNC *V. cholerae* O1 and O139 formed typical yellow colonies on F-TCBS plates, we tried to isolate cholera toxin gene-positive VBNC *V. cholerae* from environmental water samples collected in urban slum areas of Kolkata, India and succeeded in isolating *V. cholerae* O1 El Tor variant strains harboring a gene for the cholera toxin. The possible importance of VBNC *V. cholerae* O1 as a source of cholera outbreaks is discussed.

## Introduction

Cholera caused by *Vibrio cholerae* O1 and O139 continues to be a major cause of severe diarrhea, especially in developing countries (World Health Organization 2012). The infectious materials are water and food contaminated by *V. cholerae* O1 and O139. In cholera endemic areas like Kolkata, India (Nair et al. 2010) where clean drinking water is not easily available, large numbers of people use environmental water from lakes, ponds, and rivers as domestic water for washing vegetables, fish and dishes, and sometimes even as drinking water. Thus, it has been suggested that the cholera endemic continues because environmental water is contaminated by *V. cholerae* O1 and O139. However, the frequencies of isolating *V. cholerae* O1 and O139 from environmental water samples in cholera endemic areas are quite low even in epidemic seasons (Huq et al. 1990; Islam et al. 1994) and the numbers of the organisms, if isolated, are not sufficient to explain the cause of the disease (Levine et al. 1988). Moreover, between epidemic seasons *V. cholerae* O1 and O139 are very rarely isolated (Roszak and Colwell 1987).

Many investigators have reported the existence of viable but nonculturable (VBNC) *V. cholerae* O1 and O139 in environmental water in cholera endemic areas (Roszak and Colwell 1987; Alam et al. 2006), and even between epidemic seasons VBNC *V. cholerae* O1 is present in a biofilm form (Faruque et al. 2006; Alam et al. 2007). These data strongly suggest that VBNC *V. cholerae* O1 and O139 are the sources of infection in these areas. To confirm this hypothesis, we tried to isolate VBNC *V. cholerae* from environmental water samples in a culturable form and report our successful results in this article.

## Material and Methods

### Bacterial strains and culture media

*V. cholerae* O1 (N16961) and *V. cholerae* O139 (VC-280) were taken from stock cultures in the laboratory repository at the National Institute of Cholera and Enteric Diseases. The culture media used were alkaline peptone water, pH 8.8 (APW) (Eiken, Tokyo, Japan), nutrient agar (NA; Difco, Franklin Lakes, NJ) supplemented with 1% NaCl (NA) and thiosulfate citrate bile salts sucrose agar (TCBS) (Eiken).

### Culture of eukaryotic cells

HT-29 cells (human colon adenocarcinoma grade II cells) were cultured in Dulbecco's modified Eagle's medium (DMEM; catalogue number: 12800-017; Gibco Life Science, Paisley, Scotland, UK) supplemented with 1.5 g L^−1^ NaHCO_3_ (Sigma, St. Louis, MO), 3.56 g L^−1^ 4-(2-hydroxyethyl)-1-piperazine-ethanesulfonic acid (Sigma), and 10% fetal bovine serum (FBS; catalogue number 10082-147; Gibco Life Science). CHO cell (Chinese hamster ovary cells) and HeLa cells (human cervical cancer cells) were cultured in minimum essential medium (MEM; catalogue number: 61100-061; Gibco Life Science) supplemented with 10% FBS, while Caco-2 cells (human epithelial colorectal adenocarcinoma cells) were cultured in MEM supplemented with 20% FBS. T84 cell (human colonic adenocarcinoma cells) and Intestine 407 cells (human jejunum and ileum cells) were cultured in DMEM supplemented with 10% FBS. All media were supplemented with 100 *μ*g mL^−1^ streptomycin and 100 UmL^−1^ penicillin. All cell cultures were carried out in a CO_2_ incubator at 37°C under 5% CO_2_.

### Preparation of VBNC *V. cholerae*

VBNC *V. cholerae* O1 (N16961) and O139 (VC-280) were prepared as described previously (Senoh et al. 2010, 2012). Each *V. cholerae* strain was inoculated into APW and incubated at 37°C for 16 h, after which the cells were collected by centrifugation at 5000*g* for 10 min at 25°C (Heraeus Biofuge Stratos; Kendro, Langenselbold, Germany), washed twice with a VBNC microcosm buffer, comprising 1% sterile solution of artificial seawater (1% Instant Ocean; Aquarium Systems, Mentor, OH), and suspended in 200 mL of VBNC microcosm buffer in a 1-L flask to a final concentration of ∼1 × 10^8^ cells mL^−1^. The cells in VBNC microcosm buffer were incubated at 4°C in the dark without shaking. After incubation for 11 weeks, no culturable cells were observed following incubation of each VBNC microcosm buffer in APW at 37°C for 16 h. Thus, the VBNC microcosm buffer after incubation for 11 weeks contained VBNC *V. cholerae* O1 (N16961) and O139 (VC-280).

### Enumeration of culturable *V. cholerae* in the microcosm

A 0.1 mL aliquot of VBNC microcosm buffer containing VBNC *V. cholerae* was serially diluted by twofold, and 0.1 mL of each dilution was inoculated into a mixture of 0.3 mL of 1.5-fold-condensed APW and 0.05 mL of phosphate-buffered saline, pH 7.0 (PBS). After incubation at 37°C for 16 h without shaking, the turbidity of each APW was observed by the naked eye and the highest dilution of the microcosm buffer that became turbid was recorded. Assuming that one culturable cell produced turbidity by growth, the number of culturable cells per milliliter of the original microcosm buffer was calculated.

### Preparation of cell extracts of eukaryotic cells

Eukaryotic cells were cultured as described above. Confluent cells in 10-cm-diameter petri dishes were washed with PBS, scrapped off, and centrifuged at 3000*g* for 5 min at 25°C. The collected cells were resuspended in 0.5 mL of PBS, mixed with 0.1-mm-diameter glass beads, and disrupted by shaking for 90 sec using a ShakeMaster (BioMedical Science, Tokyo, Japan). After centrifugation of each mixture at 20,000*g* for 5 min at 4°C, the supernatant was passed through a 0.20-*μ*m membrane filter (EMD Millipore Corporation, Billerica, MA). The obtained cell extract without further dilution (∼0.5 mL) was designated a solution of a factor converting VBNC to a culturable state (FCVC).

### Examination of *V. cholerae* conversion with FCVC solution

A 0.1-mL aliquot of a VBNC *V. cholerae* preparation and 0.05 mL of FCVC were added to 0.3 mL of 1.5-fold-condensed APW and incubated at 37°C for 16 h without shaking. When the APW became turbid, a 0.1-mL aliquot was inoculated onto TCBS plates and incubated at 37°C for 16 h. The yellow colonies that appeared were inoculated onto NA plates and incubated at 37°C for 16 h. The serotypes of the colonies on NA plates were confirmed by appropriate typing sera (Denka, Tokyo, Japan).

### Dialysis of FCVC

A 0.5-mL aliquot of FCVC was placed in a dialysis bag (SnakeSkin®; MWCO: 10K, Takara Bio Inc., Shiga, Japan) and dialysis was carried out against 2 L of 20 mmol/L Tris-HCl (PH 8.5) at 4°C for 16 h.

### Treatment of FCVC with proteinase K

A 1-*μ*L aliquot of 25 mg mL^−1^ of Proteinase K (Sigma) in PBS was added to 98 *μ*L of FCVC and incubated at 37°C for 1 h. To inactivate the proteinase K activity for further assays with VBNC *V. cholerae*, 1 *μ*L of 50 mg mL^−1^ proteinase K inhibitor (pefabloc SC; Roche Diagnostics, Mannheim, Germany) in PBS was added to 99 *μ*L of proteinase K-treated FCVC and incubated at 37°C for 1 h.

### Treatment with proteinase K and proteinase K inhibitor

A mixture of 1 *μ*L of 25 mg mL^−1^ of Proteinase K in PBS and 1 *μ*L of 50 mg mL^−1^ Pefabloc SC in PBS was incubated at 37°C for 1 h, followed by addition of the mixture to 98 *μ*L of FCVC, and incubated at 37°C for 1 h.

### Examination of heat stability of FCVC

Aliquots (0.1 mL) of FCVC were treated at different temperatures for various time intervals and then subjected to 10-fold serial dilution. A 0.05-mL aliquot of each FCVC dilution and 0.1 mL of VBNC *V. cholerae* preparation were added to 0.3 mL of 1.5-fold-condensed APW. After incubation at 37°C for 16 h, the turbidity of the APW was observed by the naked eye and confirmation of *V. cholerae* was carried out as described above.

### Preparation of TCBS plates with FCVC

TCBS plates with FCVC (F-TCBS plates) were prepared as follows. TCBS medium was dissolved in water and boiled in accordance with the manufacturer's instructions. When the TCBS had cooled to about 60°C, ∼20 mL was poured into a 9-cm-diameter petri dish together with 1.0 mL of previously added nondiluted FCVC from HT-29 cells. The solutions were mixed thoroughly and allowed to solidify.

### Collection of environmental water samples

**Figure 1:**
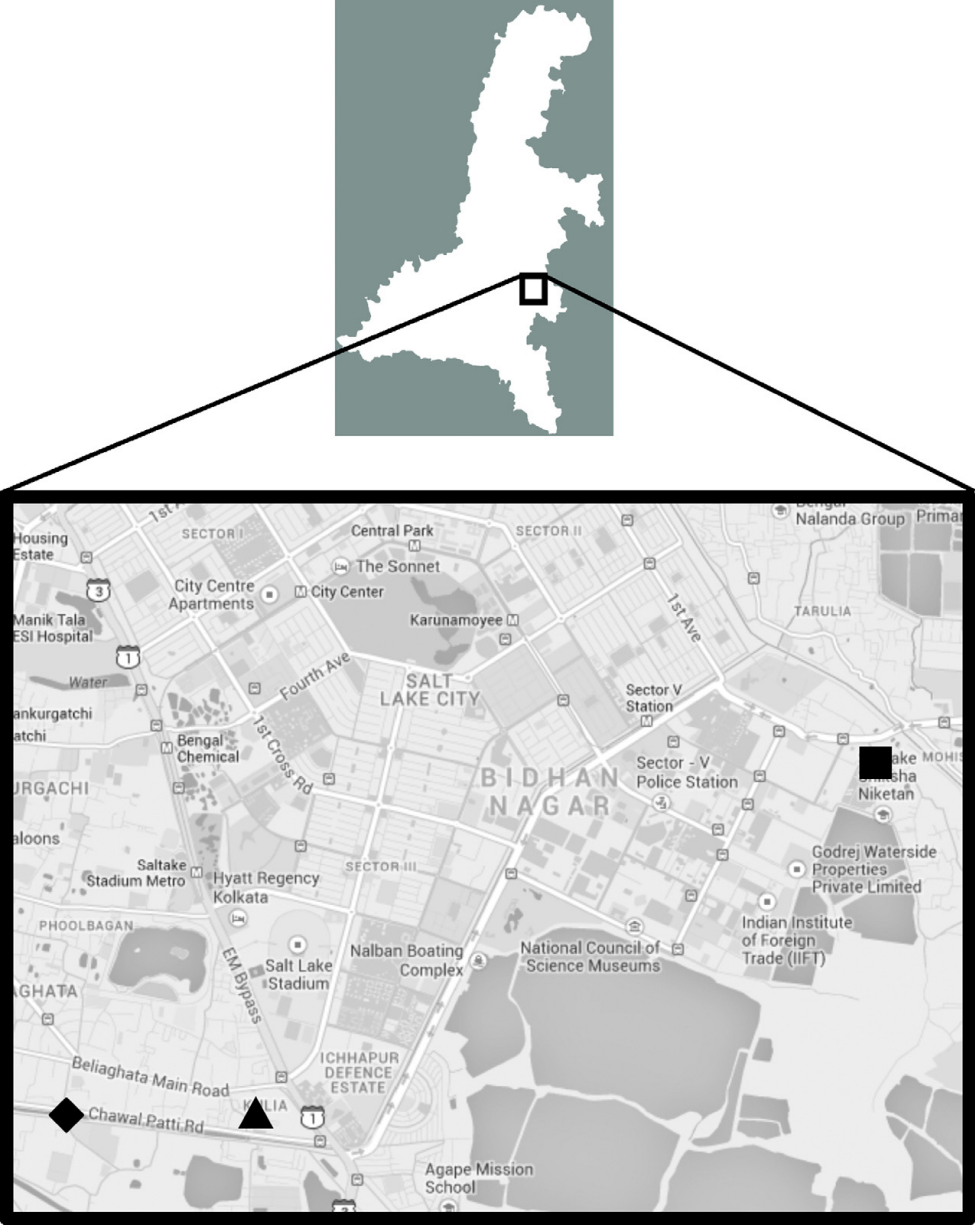
Map (from Google Maps) showing the sampling points in Kolkata, India. (▪), Points A and B; (▴), Point C; (♦), Point D.

Four sampling points were identified in urban slum areas of Kolkata (Fig. 1). Points A and B were different sites in a small pond of about 2500 m^2^. Point C was at a backwater in another slum area. Point D was at a riverside. At each of the sampling points, 50 mL of water was collected twice a month, with at least 2-week intervals, for 1 year (October 2010–September 2011).

### Examination of environmental water samples for existing VBNC *V. cholerae*

Aliquots (0.1 mL) of the collected environmental water samples were inoculated directly on both F-TCBS and TCBS plates and incubated at 37°C for 16 h. When the total numbers of colonies formed on the plates were <10, the water samples were condensed by 10-fold. Aliquots (0.1 mL) of the condensed samples were then used for direct plating on F-TCBS and TCBS plates.

### Polymerase chain reaction

To identify the environmental isolates as cholera toxin-producing *V. cholerae* polymerase chain reaction (PCR) for a *V. cholerae-*specific gene, *ompW* and a cholera toxin-specific gene, *ctx* was carried out as described previously (Shirai et al. 1991; Nandi et al. 2000). Mismatch amplification mutation assay (MAMA) PCR was carried out as described by Morita et al. (2008).

## Results

### Preparation of VBNC *V. cholerae*

**Figure 2:**
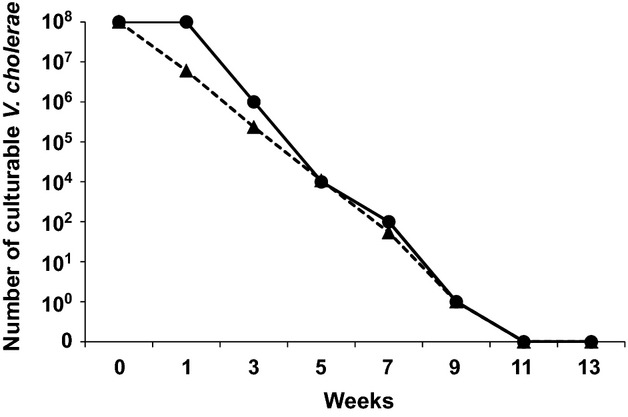
Reduction in the number of culturable cells of *Vibrio cholerae* in a VBNC microcosm buffer. Approximately 1 × 10^8^ cells mL^−1^ of *V. cholerae* O1 (N16961) and *V. cholerae* O139 (VC-280) in VBNC microcosm buffer were incubated at 4°C in the dark without shaking. The number of culturable cells in each VBNC microcosm buffer was determined in duplicate. Representative results of three experiments are presented. (•), *V. cholerae* O1 (N16961); (▴), *V. cholerae* O139 (VC-280).

A VBNC microcosm buffer containing ∼1 × 10^8^ cells mL^−1^ of either *V. cholerae* O1 (N16961) or *V. cholerae* O139 (VC-280) was incubated at 4°C in the dark without shaking. Culturable cells in each VBNC microcosm buffer were enumerated periodically as described in the Material and Methods. As shown in Figure 2, no culturable cells of either *V. cholerae* O1 (N16961) or O139 (VC-280) were observed after incubation for 11 weeks. Furthermore, 0.1-mL aliquots of these VBNC microcosm buffers did not produce any colonies on either NA or TCBS plates. In further experiments, these VBNC microcosm buffers were used as VBNC *V. cholerae* O1 (N16961) and *V. cholerae* O139 (VC-280).

### Conversion of VBNC *V. cholerae* O139 to a culturable state by extracts of eukaryotic cells

Cell extracts of various eukaryotic cells converted VBNC *V. cholerae* O139 (VC-280) to a culturable state (Table 1). In this experiment, twofold serially diluted cell extracts of various eukaryotic cells were added to APW. After inoculation of VBNC *V. cholerae* O139 (VC-280) into the APW, incubation was carried out at 37°C for 16 h without shaking. The conversion was confirmed by turbidity of the APW. Growth of VBNC *V. cholerae* O139 (VC-280) was observed after addition of the cell extracts of various eukaryotic cells, while no growth of VBNC *V. cholerae* O139 (VC-280) was observed without addition of the cell extracts. Among the eukaryotic cells examined, the cell extract of HT-29 cells showed the highest conversion activity. These results indicate that the cell extracts of eukaryotic cells contained a factor that converts VBNC *V. cholrae* into a culturable state. We named this factor FCVC.

**Table 1:** Conversion of VBNC *Vibrio cholerae* O139 (VC-280) into culturable state by cell extract of various eukaryotic cells.

Source of cell extract	HT-29	T84	Caco-2	Intestine 407	HeLa	CHO
With cell extract of eukaryotic cells	1:10^1^	1:4	1:1.8	1:2.5	1:0.6	1:4
Without cell extract of eukaryotic cells	–2	–	–	–	–	–

Caco-2, human epithelial colorectal adenocarcinoma cells; HeLa, human cervical cancer cells.

1 Highest dilution of cell extract at which VBNC *V. cholerae* O139 (VC-280) was converted into culturable state. Each experiment was repeated five times.

2 No conversion was observed. Each experiment was repeated five times.

### Some properties of FCVC

Some properties of the VBNC *V. cholerae* conversion activity of FCVC from HT-29 are presented in Table 2. After FCVC was dialyzed in a dialysis bag (SnakeSkin®; MWCO: 10K) the conversion activity was retained in the dialysis bag, indicating that the molecular weight of FCVC was more than 10,000. Regarding the effect of proteinase K on the VBNC *V. cholerae* conversion activity of FCVC from HT-29 cells, FCVC lost its conversion activity after the proteinase treatment, and this effect was inhibited by treatment with proteinase K inhibitor.

**Table 2:** Some properties of FCVC from HT-29 cells.

Dialysis	
Before	1:16^1^
After	1:16
Treatment with proteinase K	
None	1:16
Treatment with proteinase	–2
Treatment with proteinase and proteinase inhibitor	1:16

1 Highest dilution of FCVC at which VBNC *Vibrio cholerae* O139 (VC-280) was converted into a culturable state.

2 No conversion was observed.

**Figure 3:**
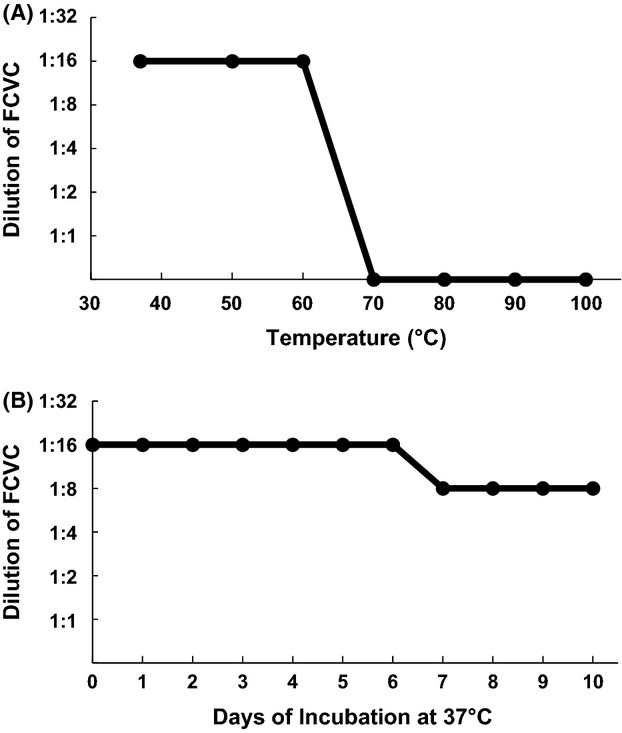
Heat stability of FCVC from HT-29 cells. (A) Aliquots (0.1 mL) of FCVC from HT-29 cells were treated at various temperatures for 5 min and the treated samples were subjected to 10-fold serial dilution. An aliquot (0.05 mL) of each diluted sample was added to 0.3 mL of 1.5-fold-condensed APW, to which 0.1 mL of VBNC *Vibrio cholerae* O139 (VC-280) had been added. After incubation at 37°C for 16 h, turbidity was observed by the naked eye and recorded. When the alkaline peptone water (APW) became turbid, it was plated on NA plates and randomly selected colonies were confirmed to be *V. cholerae* O139 by serotyping. Each experiment was repeated three times. (B) Aliquots (0.1 mL) of FCVC from HT-29 cells were incubated at 37°C for the indicated time periods and the incubated samples were subjected to 10-fold serial dilution. Further experimental procedures were as described in (A).

The heat stability of the VBNC *V. cholerae* conversion activity of FCVC from HT-29 cells was examined and the results are shown in Figure 3A. FCVC was stable during incubation at less than 60°C for 5 min, but lost its activity after incubation at 70°C or higher. As shown in Figure 3B, when FCVC was incubated at 37°C, it retained its activity for 6 days. After 7 days, the activity decreased to one-half, but subsequently remained the same for at least for additional 4 days.

These results suggest that the FCVC is a heat-stable protein with a molecular weight of more than 10,000.

### Conversion of VBNC *V. cholerae* on TCBS plates with FCVC

**Figure 4:**
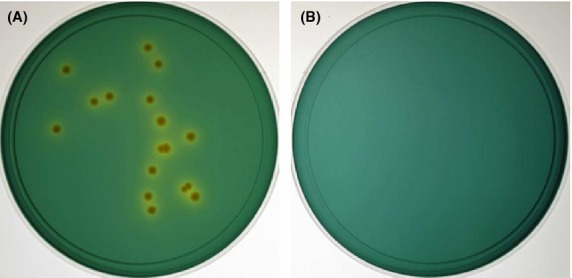
Colony formation of VBNC *Vibrio cholerae* O139 on F-TCBS plates. Aliquots (0.1 mL) of VBNC *V. cholerae* O139 (VC-280) were plated on F-TCBS (A) and TCBS (B) plates, and incubated at 37°C for 16 h.

We examined whether VBNC *V. cholerae* O139 (VC-280) was converted to a culturable form on F-TCBS plates. As shown in Figure 4A, VBNC *V. cholerae* O139 formed yellow colonies on F-TCBS plates, while no colonies appeared on TCBS plates without FCVC (Fig. 4B). These results indicated that VBNC *V. cholerae* O139 was converted to a culturable state on F-TCBS plates, and thus formed colonies.

### Isolation of VBNC *V. cholerae* from environmental water samples

Using F-TCBS plates, we tried to isolate VBNC *V. cholerae* O1 and O139 from environmental water samples collected in urban slum areas of Kolkata, India. As shown in Table 3, the number of colonies that appeared on F-TCBS plates (total: 14,978) was significantly higher than the number that appeared on TCBS plates without FCVC (total: 10,669). As yellow colonies appeared on both F-TCBS and TCBS plates, typical *Vibrio*-like colonies observed by the naked eye were subjected to PCR for a *V. cholerae-*specific gene, *ompW*. About 19 times more *ompW-*positive strains were identified from colonies on F-TCBS plates (total: 1894) compared with the number from TCBS plates without FCVC (total: 101). All *ompW-*positive strains were analyzed for whether they harbored a cholera toxin gene, *ctx*. A total of 120 *ctx-*positive strains were identified from F-TCBS plates, while only six were identified from TCBS plates without FCVC. These *ctx*-positive *V. cholerae* strains were only detected at sampling points A and B. All *ompW-*positive, *ctx-*positive *V. cholerae* strains were confirmed to be serotype O1, biotype El Tor variant by their polymyxin B sensitivity and to harbor classical *ctxB* by MAMA-PCR.

**Table 3:** Number of VBNC *Vibrio cholerae* isolated from environmental water samples.

FCVC	Total number of colonies^1^	Total number of yellow colonies	Total number of typical *Vibrio*-like colonies	Total number of *ompW-*positive strains	Total number of *Ctx-*positive strains
Point A					
+	4359	1540	1053	626	36
−	2976	899	52	37	4
Point B					
+	4292	1483	1149	750	84
−	2961	1159	58	28	2
Point C					
+	3199	1158	678	341	0
−	2562	593	92	21	0
Point D					
+	3128	953	504	177	0
−	2170	520	37	15	0
Total					
+	14,978	5134	3384	1894	120
−	10,669	3171	239	101	6

1 Data are the sums of the numbers observed for all environmental samples collected twice a month for 1 year.

**Figure 5:**
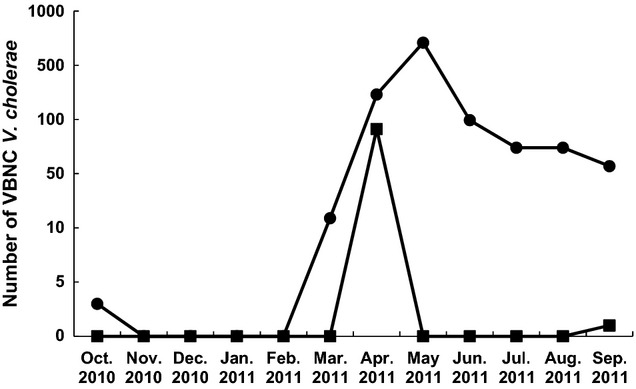
Monthly isolation of VBNC *Vibrio cholerae* from environmental water samples in slum areas of Kolkata. The sums of the numbers of *ompW-*positive*V. cholerae* (•) and *ompW-*positive, *ctx-*positive *V. cholerae* O1 (▪) observed during each month were plotted.

The seasonal distribution of VBNC *V. cholerae* strains isolated from environmental water samples is shown in Figure 5. The *ompW-*positive VBNC *V. cholerae* strains were isolated throughout the year, although there was a peak of isolation from April to June, with the highest isolation in May. On the other hand, most of the *ompW-*positive, *ctx-*positive VBNC *V. cholerae* strains were isolated in April. In September, only one *ompW-*positive, *ctx-*positive VBNC *V. cholerae* strain was isolated.

## Discussion

The VBNC state in bacteria is defined as bacteria that remain viable but are unable to grow or divide on, or in, routinely used bacterial media. As the first report by Xu et al. (1982) on VBNC *Escherichia coli* and *V. cholerae*, the existence of VBNC states in the environment of various bacteria, such as *V. cholerae (*Brayton et al. 1987; Aulet et al. 2007), *E. coli* (Garcia-Armisen and Servais 2004; Lleo et al. 2005; Zimmerman et al. 2009), *Enterococcus faecalis* (Lleo et al. 2005), and *Arcobacter* spp. (Fera et al. 2010), has been reported.

Conversion of VBNC bacteria to a culturable state has been reported under several conditions, such as temperature upshift (Wai et al. 1996; Gupte et al. 2003), incubation in phosphate buffer (Dukan et al. 1997), supplementation with catalase or sodium pyruvate (Mizunoe et al. 2000), addition of heat-stable autoinducer of growth (Reissbrodt et al. 2002), addition of resuscitation-promoting factor (Panutdaporn et al. 2006), and presence of *Acanthamoeba castellannii* (Steinert et al. 1997). However, none of these conditions succeeded in converting VBNC bacteria from environmental samples to a culturable state. Only recently, Faruque et al. (2006) and Alam et al. (2007) reported successful isolation of VBNC *V. cholerae* from environmental water samples by passage through rabbit ileal loops.

Previously, we reported the conversion of VBNC *V. cholera* and other enteric bacteria into a culturable state by coculture with several eukaryotic cell lines including HT-29 (Senoh et al., 2010; Senoh et al. 2012). In this study, we demonstrated that a factor converting VBNC bacteria into a culturable state (FCVC) existed in cell extracts of various cultured eukaryotic cells. As FCVC was resistant to heating at 60°C, we prepared TCBS plates containing FCVC (F-TCBS plates). When VBNC *V. cholerae* O139 (VC-280) was inoculated on F-TCBS plates, colonies of *V. cholerae* O139 were formed, suggesting that the VBNC bacteria were converted into a culturable state. By using F-TCBS plates, we successfully isolated VBNC *V. cholerae* from environmental water samples in urban slum areas of Kolkata, India. Quite large numbers of *V. cholerae* O1 strains carrying the *ctx* gene formed typical yellowish colonies on F-TCBS plates. Not only during the cholera epidemic season (April), but also between epidemic seasons (September and October), VBNC *V. cholerae* O1 was isolated from environmental water samples, although the frequency of its isolation between epidemic seasons was much lower than that during the epidemic season.

The evidence presented in this study that *ctx*-positive VBNC *V. cholerae* O1 exists in environmental water within cholera endemic areas strongly supports the hypothesis that the aquatic environment of cholera endemic areas is the source of cholera outbreaks. Further systematic epidemiology of the geographical and seasonal distributions of *ctx-*positive VBNC *V. cholerae* O1 and O139 in the environment of cholera endemic areas like Kolkata using F-TCBS plates will strengthen the above hypothesis.
